# A Review of the Progress of Thin-Film Transistors and Their Technologies for Flexible Electronics

**DOI:** 10.3390/mi12060655

**Published:** 2021-06-02

**Authors:** Mohammad Javad Mirshojaeian Hosseini, Robert A. Nawrocki

**Affiliations:** School of Engineering Technology, Purdue University, West Lafayette, IN 47907, USA; mmirshoj@purdue.edu

**Keywords:** TFT, flexible TFT, total thickness, bending radius (radii), oxides, amorphous Silicon (a-Si), Polycrystalline Silicon (Poly-Si), carbon nanotube (CNT), organic semiconductors

## Abstract

Flexible electronics enable various technologies to be integrated into daily life and fuel the quests to develop revolutionary applications, such as artificial skins, intelligent textiles, e-skin patches, and on-skin displays. Mechanical characteristics, including the total thickness and the bending radius, are of paramount importance for physically flexible electronics. However, the limitation regarding semiconductor fabrication challenges the mechanical flexibility of thin-film electronics. Thin-Film Transistors (TFTs) are a key component in thin-film electronics that restrict the flexibility of thin-film systems. Here, we provide a brief overview of the trends of the last three decades in the physical flexibility of various semiconducting technologies, including amorphous-silicon, polycrystalline silicon, oxides, carbon nanotubes, and organics. The study demonstrates the trends of the mechanical properties, including the total thickness and the bending radius, and provides a vision for the future of flexible TFTs.

## 1. Introduction

Physically flexible electronics enable frontier technologies to provide novel ways to interact with the physical world and open the doors toward innovative applications, such as wearable devices [1,2,3,4], electronic skins (e-skin) [5,6,7], foldable displays [8,9], and electronic papers [10,11,12]. The advantages of flexible electronics include interfacial conformability, bendability, stretchability, and being lightweight [13]. Compared with inorganic materials (e.g., metals or oxides) biocompatible organic semiconductors can be safely used for cutaneous and sub-cutaneous applications without causing harmful side effects [14].

**Figure 1:**
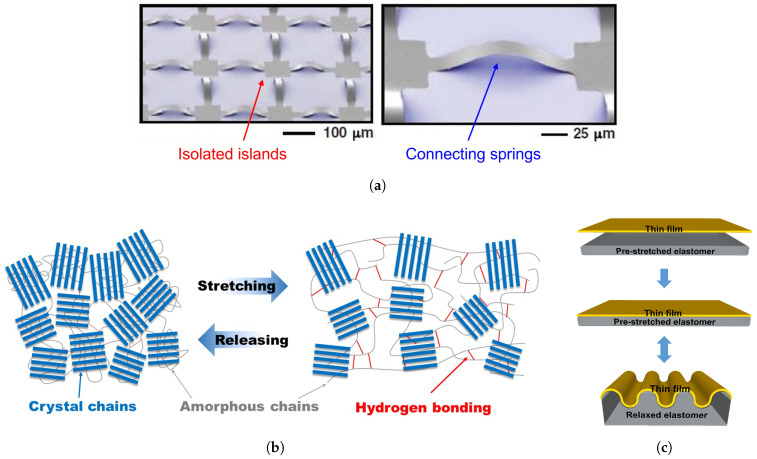
Three main approaches to physically flexible electronics; (**a**) rigid islands connected with micro-springs [5], (**b**) intrinsically stretchable materials, and (**c**) ultra-thin electronics laminated on soft and stretchable substrates. Reproduced with permission.

There are three main approaches toward flexibility in electronic devices. The first approach (Figure 1a) relies on the fabrication of distributed components on individual “islands” that are electrically and mechanically connected with individual “springs”. The main benefits of this approach are the use of high performance inorganic electronics [5]. However, a high fabrication cost is the main drawback. The second method (Figure 1b) centers around the use of intrinsically flexible and stretchable materials (Young’s modulus in the range of MPa) that are typically organic. The organic materials bear a strain over 100%; however, the intrinsically flexible materials suffer from relatively poor electrical properties (e.g., low carrier mobilities, high OFF current, and high threshold voltage), poor device stability, and restricted choice of materials [15].

The third alternative (Figure 1c) is based on the use of non-stretchable, hard, but ultra-thin materials (Young’s modulus in the range of GPa). Here, a thin film (typically few micrometers or less) is laminated on a pre-stretched elastomer, which is then allowed to relaxed. The ultra thin film buckles due to the differences in Young’s moduli. Subsequently, the structure can be repeatedly stretched up to the initial limit [16]. This method benefits from higher, full electronic interconnectivity, compared to isolated islands. However, the electronic materials used typically exhibit carrier mobilities that are orders of magnitude less compared with crystalline silicon [17].

In this review, we concentrate on the ultra-thin film approach to flexible electronics. The trends in other approaches, namely microspring-connected islands and intrinsically stretchable materials, are discussed elsewhere.

**Figure 2:**
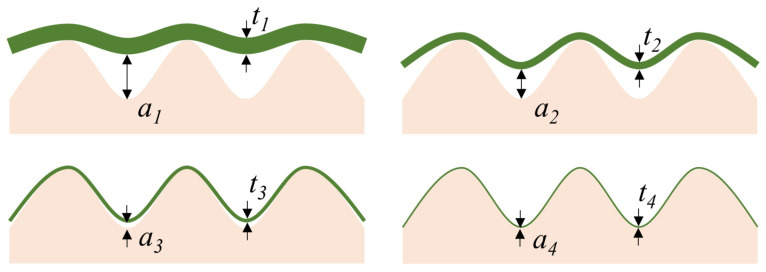
Reduction of the total film thickness reduces air gaps and improves the conformal contact between the film and the substrate.

The definition of flexibility varies across applications, from rolling in solar cells to conforming to human skin as an electrode (Figure 2) [18]. However, the bendability of a thin film is defined by its bending stiffness, which is mainly the function of two factors, the Young’s modulus and the total thickness of the thin film. A thinner film (smaller magnitude of $a_{i}$) or smaller Young’s modulus results in a more conformal film to the underlying substrate (smaller magnitude of $t_{i}$); in general, $a_{1}$ > $a_{2}$ > $a_{3}$ > $a_{4}$ results in $t_{1}$ > $t_{2}$ > $t_{3}$ > $t_{4}$ [19]. As the material choice is often dictated by technological constraints via the choice of the semiconducting material, a reduction of the Young’s modulus is often difficult or impossible. Hence, engineers often rely on reducing the total film thickness to improve the conformability.

Thin film transistors are essential devices among flexible electronic components. They are widely used in applications, such as flexible displays [20,21,22], intelligent textiles [23], and conformable radio frequency identification devices (RFID) [24]. It is the choice of the semiconductor material that defines the device technology as well as its manufacturing constraints (e.g., the choice of substrate, fabrication equipment) and mechanical (i.e., flexibility) and electrical (i.e., carrier mobilities) characteristics.

Hydrogenated amorphous silicon (a-Si:H) Thin-Film Transistors (TFTs) have been considered as a driving element in flexible displays [8,9] due to the low temperature (<300 °C) of the fabrication process and better uniformities of TFT properties compared to polycrystalline silicon (Poly-Si) TFTs [25,26]. However, instability under gate-biased stress restricts their applications in flexible devices [27].

Low-Temperature Poly-Silicon (LTPS) is a promising candidate for high frame rates and high-resolution flexible displays due to the high mobility and stable characteristics compared to a-Si:H [28]. Nevertheless, the higher temperature of Poly-Si crystallization compared with the glass transition temperature of plastic limits LTPS realization [29].

Amorphous oxide semiconductors, such as amorphous Indium Gallium Zinc Oxide and Zinc Tin Oxide, have been studied intensively due to their higher stability and better mobility compared with a-Si:H and superior uniformity over larger substrates compared to LTPS [30]. However, the higher carrier concentration of amorphous oxide semiconductors lowers the ON/OFF ratio compared to other semiconductors [31].

Single-Wall Carbon Nanotube (SWCNT) semiconductors offer flexibility, transparency, low-cost, and room temperature fabrication, as well as high intrinsic mobility [32]. Despite all the advantages, SWCNT TFTs suffer from a complex and expensive fabrication processes, uncertainty in the device uniformity, and reproducibility issues [33,34].

Organic Thin-Film Transistors (OTFTs) have demonstrated relatively low fabrication temperature (<120 °C), lower fabrication costs comparing to inorganic materials, alternative fabrication methods, such as inkjet printing, and remarkable flexibility [35]. However, organic semiconductors suffer from poor electrical properties and stability [36].

Here, we review the progress of the total thickness and bending radius of five categories of TFTs based on semiconductors consisting of a-Si:H, Poly-Si, oxides, CNT, and organic materials over the last three decades. The electrical properties, such as the on/off ratio and mobility will also be studied. The fabrication process, device architecture, and the application of every category are also investigated.

## 2. Hydrogenated Amorphous Silicon

The first demonstration of flexible hydrogenated amorphous silicon devices dates back in 1983 when Okinawa et al. fabricated an a-Si:H solar cell on a plastic substrate [37]. Since 1986, when the first a-Si:H-based LCD appeared, bendable displays have been considered the main application of hydrogenated amorphous silicon TFTs [38]. Plasma-Enhanced Chemical Vapor Deposition (PECVD) is mainly used to deposit a-Si:H, and plasma keeps the decomposition temperature of SiH4 lower than 300 °C [39]. The mobility of TFTs using PECVD techniques reaches 0.5–1 cm^2^/Vs for electrons and approximately $10^{-2}$ cm^2^/Vs for holes [40].

However, technologies, such as Hot Wire Chemical Vapor Deposition (HWCVD), offer the mobility of 4.7 cm^2^/Vs for a-Si:H TFTs, yet such fabrication techniques are still not fully commercialized [41,42]. The typical a-Si:H TFT structure is bottom-gate, staggered, and Bach Channel Etched (BCE) which has advantages in fabrication procedures [27]. The leakage current is in the range of $10^{-12}$ A, and the long-term reliability of TFTs is lower than that of LTPS devices [43].

The a-Si:H TFT typically has an ON/OFF ratio over $10^{6}$, a threshold voltage of less than 3 V, and a sub-threshold slope less than 0.5 V/dec [44]. The threshold voltage of a-Si:H TFTs shows instability under immense stress for a prolonged time due to the creation of dangling bonds and charge trapping [45]. They have a lower mobility compared to other semiconductors, such as LTPS, a limited high speed, and large-current applications [46].

**Figure 3:**
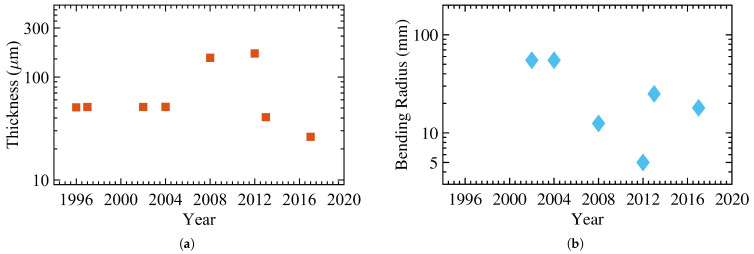
The progress of hydrogenated amorphous silicon (a-Si:H) Thin-Film Transistors (TFTs) with (**a**) the total thickness and (**b**) the bending radius.

Figure 3 demonstrates a subset of studies illustrating the progress regarding the thickness and bending radius over the last three decades [47,48,49,50,51,52,53,54]. Shen et al. reported an a-Si:H over a 50μm alkali-free glass foil in 1996. In 1997, Shen et al. demonstrated a network of a-Si:H TFT for backplanes of active-matrix liquid crystal displays using the same structure. Sturm et al. realized and characterized a network of hydrogenated amorphous silicon transistors distributed across a 50μm polyimide (PI) substrate in the following years.

**Figure 4:**
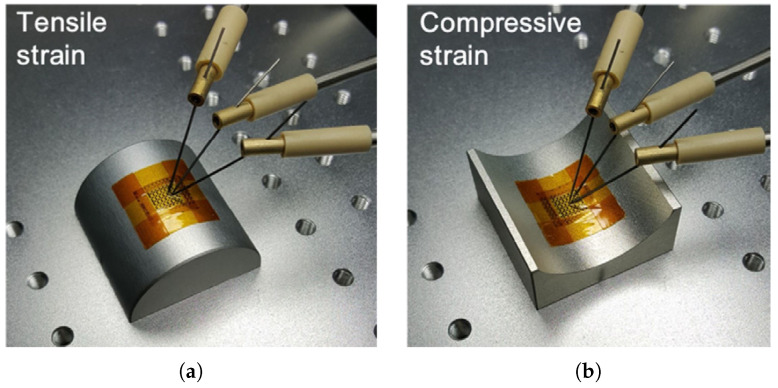
An example of physically flexible a-Si:H transistors under (**a**) tensile and (**b**) compressive strain [54]. Reproduced with permission.

The bending radius is estimated at 55 mm. Jang et al. presented a flexible TFT device based on a-Si:H that showed the same electrical characteristics as rigid devices in 2008. They fabricated the device over a 150μm stainless steel foil, and the bending radii reached 12.5 mm. In 2012, Fruehauf et al. introduced the most bendable TFTs based on a:Si:H with a bending radius of 5 mm over a 75μm glass foil. In the following years, TFTs became thinner, as low as 26.22μm; however, the lowest bending radius remained unchanged. Table 1 lists the main electrical and mechanical characteristics of amorphous silicon devices with Figure 4 showing an example of flexible a-Si:H TFTs.

## 3. Polycrystalline Silicon

In 1984, Harbeke et al. showed that poly-Si had higher stability with lower roughness compared with a-Si [55]. However, the deposition process (Low-Pressure Chemical Vapor Deposition followed by thermal recrystallization) temperature reached 1000 °C, which made using plastic substrates impossible [56]. The introduction of the XeCl excimer laser in 1986 reduced the maximum temperature of the process to less than 260 °C, but the temperature was still higher than the melting temperature of most plastic substrates [57].

In the following years, the crystallization methods, such as Excimer Laser Annealing (ELA), Solid Phase Crystallization (SPC), and Sequential Lateral Solidification (SLS) opened the doors to deploying plastic substrates [58]. The Low-Temperature Polycrystalline silicon TFTs offers carrier mobility of 20 to 500 cm^2^/Vs and the leakage current of $10^{-12}$ A makes it a promising candidate for bendable display industries [59]. Furthermore, the higher thermal conductivity of LTPS (32 W/mK) protects it from self-induced thermal degradation, compared to other semiconductors, such as metal oxide semiconductors with a thermal conductivity of 1.4 W/mK [60,61].

In-grain defects and nonuniform distribution of the grain boundaries along the device channel lead to the variation in threshold voltages from device to device over the same substrate and limits in deploying LTPS TFTs for analog circuits. Moreover, the process temperature is still challenging for the majority of plastic substrates [62].

**Figure 5:**
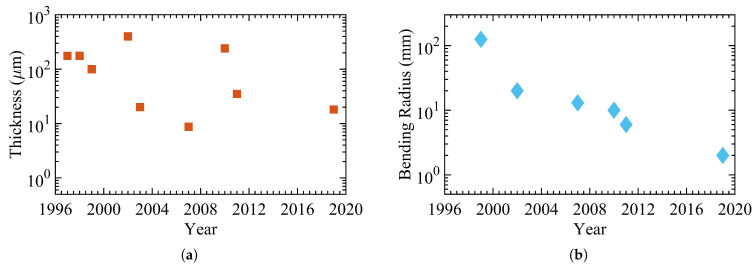
The progress of LTPS TFTs with (**a**) the total thickness and (**b**) the bending radius.

The downward trend of the total thickness and bending radius of LTPS TFTs since the 1990s is shown in Figure 5 [63,64,65,66,67,68,69,70,71]. Sigmon et al. reported one of the earliest LTPS devices on a 175-μm polyester substrate in 1997. The following year, King et al. demonstrated a device with the same thickness fabricated on a Polyethylene Terephthalate (PET) substrate that showed a higher ON/OFF ratio. Omata et al. recorded the highest mobility for a flexible device in 1999 using a 100-μm stainless steel substrate. The bending radius for the device was estimated at 20 mm. Three years later, in 2002, Shimoda et al. developed a flexible Liquid Chrystal Display (LCD) based on LTPS TFTs. The device was fabricated on a 400-μm Polyethersulfone (PES) substrate, and the bending radius was 20 mm.

Fonash et al. introduced a fabrication method based on a separation technique to keep the mobility as high as 174 cm^2^/Vs, while the thickness decreased to 20μm. In 2007, Fortunato et al. reported the thinnest LTPS TFT fabricated on an 8-μm PI bent to 13 mm. Kim et al. from Samsung R&D center reported a 2.8-inch flexible display based on LTPS TFT on a 240-μm substrate. The bending radius of the display reached 10 mm.

**Figure 6:**
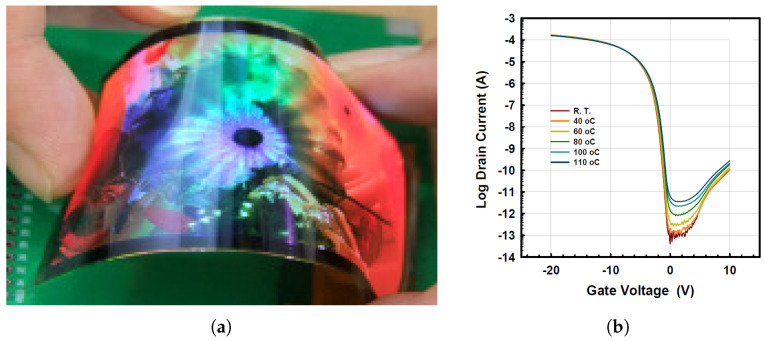
An example of one of the original physically flexible LTPS TFTs [69]. (**a**) Photograph of a flexible substrate with TFTs and circuits. (**b**). Transfer curve of a since LTPS device. Reproduced with permission.

Bearzotti et al. demonstrated a network of LTPS inverters beside three sensory units in 2011. The device was fabricated on an 8-μm PI substrate, and the bending radius was as low as 6 mm. The most bendable LTPS TFT was shown in 2019 by Park with the reported thickness of 18.05 μm and bending radii of 2 mm. Table 2 summarizes the main electrical and mechanical properties of the polycrystalline silicon devices shown in Figure 5, with Figure 6 showing an early example of flexible LTPS TFTs.

## 4. Oxides

Since the first demonstration of amorphous Indium-Gallium-Zinc-Oxide (a-IGZO) in 2003–2004 [72,73], oxide semiconductors have attracted a significant amount of attention in flexible electronics areas due to their optical transparency, high carrier mobility, and lower process temperature compared with a-Si [74]. There are four more oxide semiconductors in addition to a-IGZO that have been deployed to realize flexible oxide TFTs, namely Zinc Oxide (ZnO), Zinc Tin Oxide (ZTO), Indium Zinc Oxide (IZO), and Zinc Indium Tin Oxide (ZITO) [75]. Sputtering is the dominant technology to deposit oxides [76]; however, other methods, such as printing [77], spin coating [78], and Pulsed Laser Deposition (PLD) [79], have also been reported.

The application of metal oxide TFTs has been reported mostly for bendable/paper displays [80,81], wearable sensors [82], flexible memories [83,84], and energy storage [85]. While the maximum reported mobility for oxides surpasses 150 cm^2^/Vs, it is commonly in the range of 1 to 100 cm^2^/Vs [86]. The leakage current for oxide TFTs is as low as $10^{-13}$ A [30]. Polyimide (PI), polycarbonate (PC), polyethylene terephthalate (PET), and polyethylene naphthalate (PEN) are the most commonly plastic substrates for oxide TFTs [87], while other plastics, such as polydimethylsiloxane (PDMS) [88], polyurethane (PU) [89], and thin glass foil [90], are rarely used.

The oxide semiconductors are deposited at room temperature; however, the TFT processing temperature, particularly for dielectric fabrication, is in excess of 150 °C making oxide TFTs incompatible with transparent plastics, such as cyclo-olefins. The higher carrier concentration of amorphous oxide semiconductors typically offers lower ON/OFF ratios compared to other semiconductors [31].

**Figure 7:**
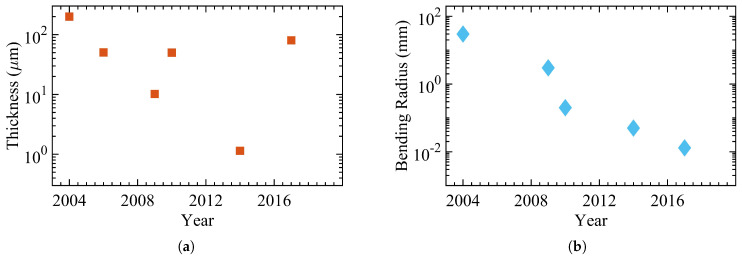
The progress of oxide TFTs with (**a**) the total thickness and (**b**) the bending radius.

Figure 7 presents the trends in oxide TFTs, demonstrating the total thickness and the bending radius since 2004 [73,91,92,93,94,95]. Hosono et al. reported one of the earliest amorphous IGZO (a-IGZO) TFTs on a 200μm PET substrate with a bending radii of 30 mm. In 2006, a ZTO TFT was fabricated by Hauschildt et al. on a stainless steel-backed PI sheet with an overall thickness of 50.56μm. Three years later, a light-emitting diode display was shown by Chung et al. based on a-IGZO TFT on a 10-μm PI. The display showed a bending radius of 3 mm. In 2010, Moon et al. introduced a flexible, solution-based oxide TFT based on Zinc oxide with a bending radius of 0.2 mm and a thickness of approximately 50μm.

**Figure 8:**
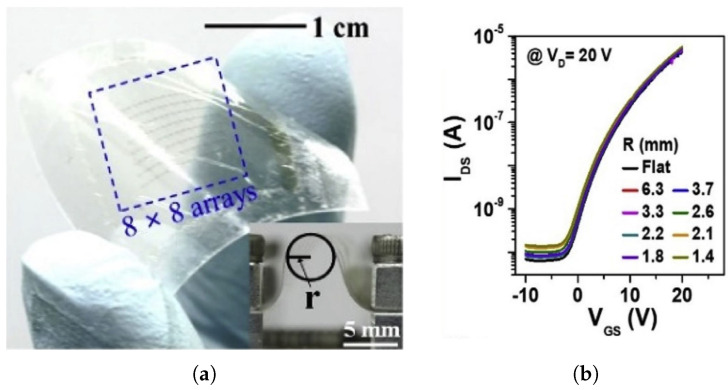
(**a**) Manual mechanical bending of an IGZO TFT film. The inset shows a zoomed view of the bent film (**b**) Transfer curve of the IGZO TFTs for different bending radii [96]. Reproduced with permission.

Tröster et al. demonstrated an ultra-flexible TFT based on a-IGZO that was capable of wrapping around a human hair with a bending radius of 50μm. The device was fabricated on the thinnest reported substrate for oxide TFTs with 1.145μm using polydimethylsiloxane (PDMS). In 2017, the most bendable oxide TFT was demonstrated with a bending radius of 13μm and a total thickness of 80.385μm. Table 3 shows the detailed properties of the oxide TFTs shown in Figure 7, with Figure 8 showing a recent example of an array of flexible oxide (IGZO) TFTs.

## 5. Carbon Nanotubes

In 1998, two separate research groups demonstrated thin-film transistors based on single and multi-wall carbon nanotubes on silicon substrates [97,98]. Rinzler et al. presented a process to deposit a SWCNT film on a flexible substrate for the first time in 2004 [99]. The properties of SWCNTs, such as small geometric size (≈1 nm), low power dissipation due to the possibility of ballistic transport, and good thermal conductivity (3500 W/mk), make single-wall carbon nanotubes a promising channel material [100,101,102]. The non-vacuum and low processing temperature of SWNTs allow for the direct deposition on flexible substrates [103].

The fabrication processes are grouped into two categories; (1) the solution process, including printing [103] and solution-based coating techniques [104], such as spray coating [105] and drop-casting [106]; and (2) dry processes, such as Chemical Vapor Deposition (CVD) [107]. Different applications have been demonstrated for flexible SWCNT TFTs, including integrated circuits [108], sensors [109,110], light-emitting diodes [111], and touch panels [112]. While the mobility of a uniform network of SWCNTs can reach as high as 10,000 cm^2^/Vs, the reported mobilities of flexible devices is limited to 10 to 100 cm${}^{2}$/Vs resulting from a random distribution of nanotubes across the channel [100]. Devices based on SWCNT channel materials suffer from poor reproducibility due to limitations over the synthesis of homogeneous structures and difficulties in the controllable formation of SWCNT assemblies over large areas [113].

**Figure 9:**
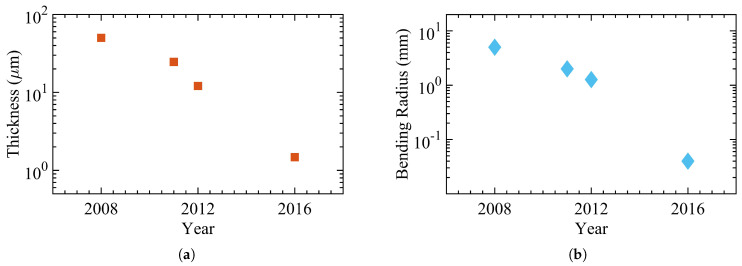
The progress of SWCNT TFTs with (**a**) the total thickness and (**b**) the bending radius.

**Figure 10:**
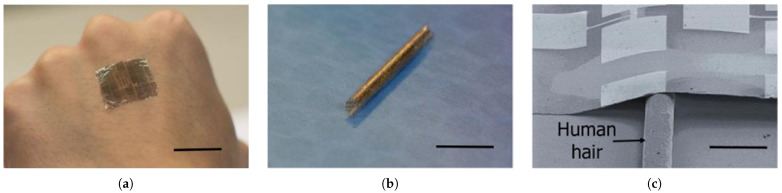
Ultrathin and flexible carbon nanotube electronic sheet, (**a**) laminated onto human skin, and (**b**) rolled-up. (**c**) SEM image of the CNT film laminated over a human hair. Scale bars are (**a**) 2 cm, (**b**) 1 cm, and (**c**) 150 μm. [117]. Reproduced with permission.

The very first flexible SWCNT TFT, fabricated on a 50μm polyimide substrate, was demonstrated by Rogers et al. in 2008. Nearly 100 transistors were implemented on a plastic substrate and showed no significant changes when bent, with the bending radius as low as 5 mm. Three years later, Javey et al. fabricated a large-scale array of SWCNT TFTs on a 24-μm PI substrate, which became stretchable by the formation of a honeycomb mesh structure made via direct laser patterning. The bending radii reached 2 mm thanks to the thinner substrate and the mesh structure. In 2012, Javey et al. presented a thinner TFT with a total thickness of 12.105-μm, which resulted in a bending radius of 1.27 mm. The thinnest and the most flexible SWCNT TFT was demonstrated in 2016 with a total thickness of 1.476μm and a bending radius of 40μm. The TFT film showed little degradation under 67% compressive strain. Inverters, and NAND and NOR gates were also realized, and showed negligible performance changes under 33% compressive strain. Figure 9 shows the trend of total device thickness and bending radius of the SWCNT TFTs [114,115,116,117]. Table 4 summarizes the main electrical and mechanical properties of the SWCNT TFTs shown in Figure 9, with Figure 10 showing a recent example of flexible CNT TFTs.

## 6. Organic Semiconductors

The first organic TFTs were demonstrated in 1986, fabricated on a silicon substrate with recorded carrier mobilities close to 10${}^{-5}$ cm^2^/Vs [118]. In 1994, the first flexible Organic Field Effect Transistor (OFET) was demonstrated, with the device fabricated on a 1.5μm thin PET substrate [119]. Anthopoulos et al. reviewed the reported mobility of fabricated OFETs over the past 30 years and showed that the maximum mobilities for p-type and n-type organic semiconductors were typically below 20 and 10 cm^2^/Vs, respectively [13]. Organic field-effect transistors are implemented with four major structures: (1) single gates, including top gate and bottom gate structures, (2) dual gates to improve charge carrier modulation, (3) vertical channel structures, and (4) cylindrical gate structures [35].

The ON/OFF ratio can be as high as 10${}^{6}$, and the leakage current can be as low as one pA [120,121]. Vacuum evaporation techniques and solution-based processing are two major deposition methods for organic materials, with the former typically providing higher carrier mobility compared with the latter [122,123,124]. However, solution-based deposition methods are beneficial for large-area electronics realization, while the process temperature is low (typically room temperature) and keeps the costs low as well [125].

Flexible OFETs are used for various applications, including displays [126], tags [127], sensors [128], wearable devices [129], integrated circuits [130], and medical devices [131]. Despite the advances in organic semiconductors, the carrier mobilities of OFETs are much lower than other technologies that limit high-speed applications. Yet, with p- and n-type organic semiconductors available, complimentary organic circuits have been shown, something that is yet to be demonstrated for other flexible inorganic technologies.

Bonfiglio et al. demonstrated the earliest OFETs fabricated on a flexible substrates. They presented various types of devices on biaxially oriented PET (known as Mylar) using Pentacene as the semiconductor. Bonfiglio continued demonstrating organic transistors, including solution processed, complimentary (p- and n-type), and transparent devices [132,133,134]. In 2010, Someya et al. reported an OFET fabricated directly on a 12.5-μm thick PI that was bendable down to 0.1 mm.

Three years later, the same group demonstrated a device with a total thickness of approximately 2 μm using dinaphtho[2,3-b:2${}^{\prime }$,3${}^{\prime }$-f]thieno[3,2-b]thiophene (DNTT) as the organic semiconductor. The device could endure 230% tensile strain, while the bending radius was as low as 5 μm. In 2016, Someya et al. demonstrated the thinnest OFET to date, with a total device thickness of 270 μm and a bending radius of 1.5 μm. The device is the thinnest and the most bendable reported TFT of any technology and material.

**Figure 11:**
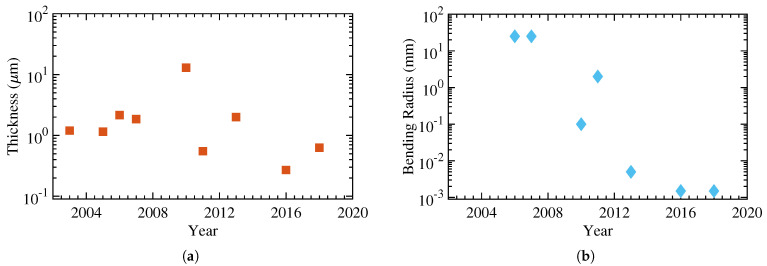
The progress of organic TFTs with (**a**) the total thickness and (**b**) the bending radius.

**Figure 12:**
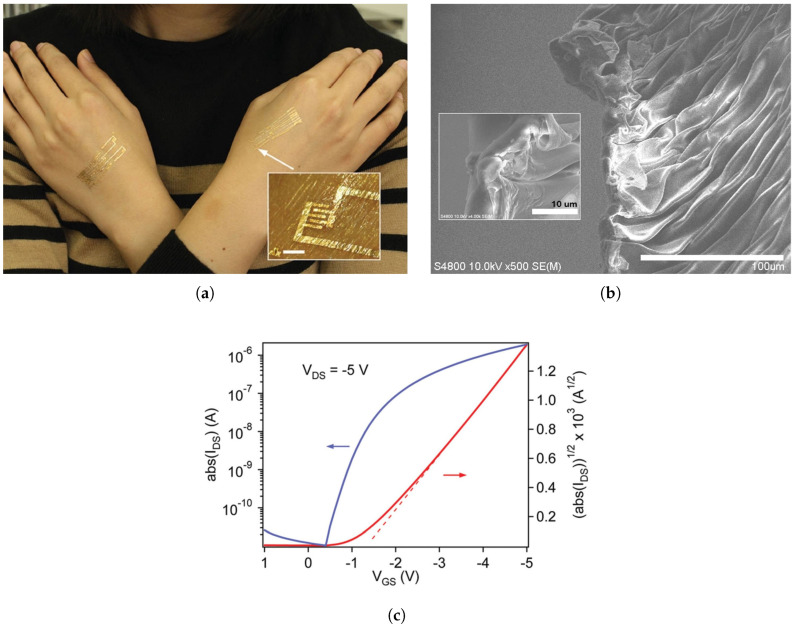
Ultrathin, flexible, and biocompatible organic electronic TFT, (**a**) shown laminated on human skin. The inset shows a zoomed view of a tactile sensor, with skin grooves visible. The scale bar is 2 mm. (**b**) SEM image indicating ultra-small film wrinkles. The scale bars are 100 μm in the main image, and 10 μm in the inset. (**c**) Transfer curve of organic TFT. [141]. Reproduced with permission.

The following year, Chan et al. fabricated OFETs using ultra-thin substrates. While the bending radius remained unchanged, the devices were shown surviving sterilization in 100 °C boiling water or steam, without much change to their electrical characteristics. Figure 11 demonstrates the information regarding total thickness and the bending radii for the papers in this section [16,135,136,137,138,139,140,141,142]. Table 5 lists the main electrical and mechanical properties as well, with Figure 12 showing an example of flexible organic semiconductor TFTs.

## 7. Comparison of Semiconducting Technologies

Different technologies offer different benefits and drawbacks. For instance, while poly-Si offers the highest mobilities of thin and flexible transistors, they lag in flexibility (expressed as bending radii) compared to other technologies. Additionally they require somewhat higher processing temperatures. CNTs offer low processing temperatures, very low bending radii, and relatively high mobilities.

**Figure 13:**
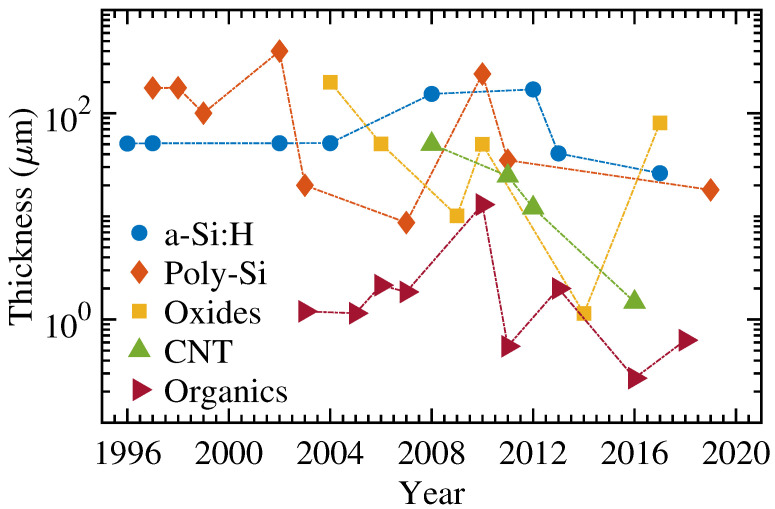
The comparison of thickness over a-Si:H, poly-Si, oxides, CNTs, and organic semiconducting technologies.

**Figure 14:**
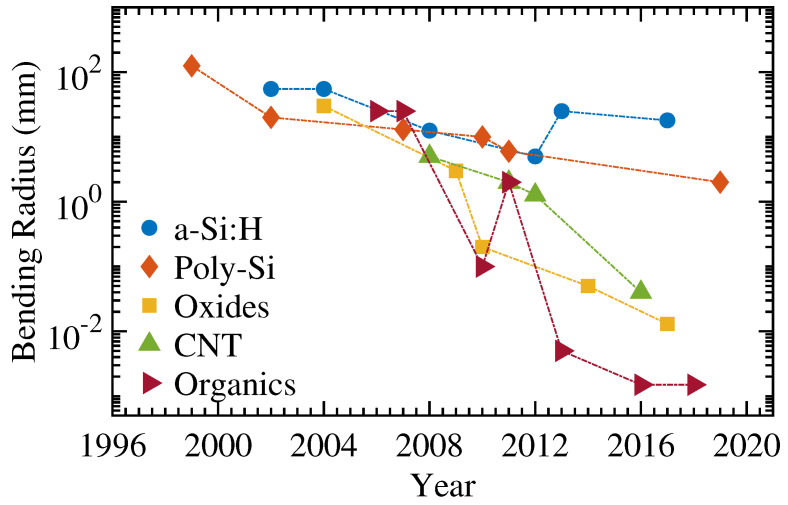
The comparison of bending radius over a-Si:H, poly-Si, oxides, CNTs, and organic semiconducting technologies.

Organic semiconductors offer the highest flexibility with the lowest bending radii. However, they offer relatively low carrier mobilities. Additionally, they are typically susceptible to environmental degradation (due to the exposure of oxygen, moisture, and UV) and, hence, need to be encapsulated. They are also the only technology that offers both p- and n-type, thus, enabling low-power complimentary electronics. Table 6, Figure 13 and Figure 14 briefly summarizes all the technologies presented in this work. The non-monotonic nature of the figures is due to the fact that new demonstrations of thicker TFTs would result devices with smaller bending radii, or vice versa.

## 8. Conclusions

Flexible electronics have recently gained a considerable amount of attention due to the evolution of new applications that require stretchable and bendable electrical circuits and components. The readers in the field certainly can benefit from an overview regarding the categories of flexible semiconductors, the dominant properties, and the progress of thickness and bendability.

This brief review draws attention to the available flexible electronic technologies and offers a comparison between them all. Flexible TFTs based on five categories (a-Si, Poly-Si, oxides, CNTs, and organic materials) were studied in detail. For every class, the thickness and bending radii trends were investigated through a selected number of works showing the meaningful progress of increases in bendability resulting from the decreases in the total device thickness, including the substrate.

The results demonstrate organic TFTs as the thinnest and the most bendable flexible devices because the Young’s modulus of organic materials is three orders of magnitude is lower than other materials. Despite the promising mechanical properties of organic materials, they suffer from poor electrical properties. However, the advances in new organic material synthesis provide a bright future for these materials.

Amorphous silicon is the dominant technology in flexible displays due to the low processing temperature and uniformity; however, the carrier mobility has restricted high-speed applications. Polycrystalline silicon, CNTs, and oxides have shown carrier mobilities as high as 100 cm^2^/Vs; however, the total thickness of devices, limited dielectric varieties, and mechanical properties of TFT layers has restricted the minimum bending radius. 

## Figures and Tables

**Table 1:** The detailed properties of the a-Si:H TFTs in Figure 3.

Publication Year	Bending Radius (mm)	Total Thickness (μm)	ON/OFF Ratio	Channel Width/Length	Mobility (cm${}^{2}$/Vs)	Substrate	Substrate Thickness (μm)	Dielectric	Reference
1996	65	50.75	$10^{4}$	1000/100	N/A	alkali-free glass foil	50	SiN${}_{x}$	[47]
1997	65	51.15	$10^{6}$	500/150	0.41	alkali-free glass foil	50	SiN${}_{x}$	[48]
2002	55	51.16	$10^{5}$	14/4	0.47	PI	50	SiN${}_{x}$	[49]
2004	55	51.26	$10^{5}$	25/10	0.42	PI	50	SiN${}_{x}$	[50]
2008	12.5	153.7	$10^{6}$	25/5	1.47	stainless steel foil	150	SiN${}_{x}$	[51]
2012	5	75	$10^{6}$	50/10	0.4	glass foil	75	SiN${}_{x}$	[52]
2013	25	40.75	$10^{7}$	80/8	0.45	PI over glass foil	40	SiN${}_{x}$	[53]
2017	18	26.22	$10^{4}$	10/10	0.031	PI	25	SiN${}_{x}$	[54]

**Table 2:** The detailed properties of the LTPS TFTs in Figure 5.

Publication Year	Bending Radius (mm)	Total Thickness (μm)	ON/OFF Ratio	Channel Width/Length	Mobility (cm${}^{2}$/Vs)	Substrate	Substrate Thickness (μm)	Dielectric	Reference
1997	N/A	175.6	>10${}^{3}$	N/A	N/A	Polyester	175	SiO${}_{2}$	[63]
1998	N/A	176.11	>10${}^{5}$	100/50	60	PET	175	SiO${}_{2}$	[64]
1999	125	>100	$10^{6}$	N/A	106	stainless steel	100	SiO${}_{2}$	[65]
2002	20	>400	N/A	10/10	63	PES	400	SiO${}_{2}$	[66]
2003	N/A	>20	$10^{8}$	N/A	174	N/A	20	SiO${}_{2}$	[67]
2007	13	8.695	>10${}^{6}$	N/A	70	PI	8	SiO${}_{2}$	[68]
2010	10	>240	$10^{8}$	N/A	124	N/A	240	SiO${}_{2}$	[69]
2011	6	35	N/A	150/10	60	PI	10	SiO${}_{2}$	[70]
2019	2	18.05	>10${}^{7}$	5/4 and 10/6	80	PI	17	SiO${}_{2}$	[71]

**Table 3:** The detailed properties of the oxide TFTs in Figure 7.

Publication Year	Bending Radius (mm)	Total Thickness (μm)	ON/OFF Ratio	Channel Width/Length	Mobility (cm${}^{2}$/Vs)	Substrate	Substrate Thickness (μm)	Dielectric	Semiconductor	Reference
2004	30	>200	$10^{3}$	200/50	6–9	PET	200	Y${}_{2}$O${}_{3}$	a-IGZO	[73]
2006	N/A	50.56	$10^{6}$	1000/80	14	stainless steel foil-backed PI	50	SiON	ZTO	[91]
2009	3	10.15	>10${}^{8}$	N/A	15.1	PI	10	SiN${}_{x}$	a-IGZO	[92]
2010	0.2	>50	10${}^{6}$	1500/120	0.39±0.03	PI	50	SiO${}_{2}$	ZnO	[93]
2014	0.05	1.145	$10^{6}$	280/80	26	Parylene	1	Al${}_{2}$O${}_{3}$	a-IGZO	[94]
2017	0.013	80.385	$10^{7}$	280/30	13.7	PDMS	80	Al${}_{2}$O${}_{3}$	a-IGZO	[95]

**Table 4:** The detailed properties of the SWCNT TFTs in Figure 9.

Publication Year	Bending Radius (mm)	Total Thickness (μm)	ON/OFF Ratio	Channel Width/Length	Mobility (cm${}^{2}$/Vs)	Substrate	Substrate Thickness (μm)	Dielectric	Reference
2008	5	>50	10${}^{5}$	5/100	80	PI	50	HfO${}_{2}$	[114]
2011	2	24.58	10${}^{4}$	250/3	20–30	PI	24	Al${}_{2}$O${}_{3}$ and SiO${}_{x}$	[115]
2012	1.27	12.105	400	3/4	55	PI	12	Al${}_{2}$O${}_{3}$ and SiO${}_{x}$	[116]
2016	0.04	1.476	10${}^{6}$	100/10	12.04	PET	1.4	Al${}_{2}$O${}_{3}$ and SiO${}_{2}$	[117]

**Table 5:** The detailed properties of the organic TFTs in Figure 11.

Publication Year	Bending Radius (mm)	Total Thickness (μm)	ON/OFF Ratio	Channel Width/Length	Mobility (cm${}^{2}$/Vs)	Substrate	Substrate Thickness (μm)	Dielectric	Semiconductor	Reference
2003	N/A	1.2	>10${}^{3}$	60,000/20	10${}^{-4}$	Mylar	0.9	Mylar	Pentacene	[135]
2005	N/A	1.15	N/A	210,000/70	$5\times 10^{-4}$	Mylar	0.9	Mylar	Pentacene	[136]
2006	25	2.15	N/A	5000/25	N/A	Mylar	1.9	Mylar	Pentacene	[137]
2007	25	1.85	N/A	5000/25	N/A	Mylar	1.6	Mylar	Pentacene	[138]
2010	0.1	13	$10^{7}$ and $10^{5}$	500/50	0.5 and 0.1	PI	12.5	AlO${}_{x}$	Pentacene and F16CuPc	[139]
2011	2	0.55	>10${}^{3}$	5000/5	0.04±0.02, 0.03±0.02, and 0.03±0.01	Parylene	0.4	Parylene	Pentacene, TIPS, and N1400	[140]
2013	0.005	2	>10${}^{7}$	500/40	3	PEN	1.2	AlO${}_{x}$	DNTT	[16]
2016	0.0015	0.27	$10^{5}$	1000/100	0.34	Parylene	0.06	Parylene	DNTT	[141]
2018	0.0015	0.63	>10${}^{5}$	35	4.16±0.73	PAN	0.18	Al${}_{2}$O${}_{3}$	DPh-BBTNDT	[142]

**Table 6:** Comparison of semiconducting technologies.

Technology	Semiconductor	Total Thickness (μm)	Bending Radius (mm)	Mobility (cm${}^{2}$/Vs)	Process Temperature (°C)	Reference
a-Si:H		26.22	18	0.031	250	[54]
Poly-Si		18.05	2	80	230	[71]
Oxides	IGZO	>3	1.4	0.8	300	[96]
CNT		1.476	0.04	12.04	90	[117]
Organics	DNTT	0.27	0.0015	0.34	120	[141]
